# Novel treatments for rare rheumatologic disorders: analysis of the impact of 30 years of the US orphan drug act

**DOI:** 10.1186/s13023-016-0443-x

**Published:** 2016-05-12

**Authors:** Thomas Lutz, Anette Lampert, Georg F. Hoffmann, Markus Ries

**Affiliations:** Center for Pediatric and Adolescent Medicine/Pediatric Rheumatology, and Center for Rare Diseases, Heidelberg University Hospital, Im Neuenheimer Feld 430, 69120 Heidelberg, Germany; Department of Clinical Pharmacology and Pharmacoepidemiology, Heidelberg University Hospital, Im Neuenheimer Feld 410, 69120 Heidelberg, Germany; Cooperation Unit Clinical Pharmacy, Heidelberg University Hospital, Im Neuenheimer Feld 410, 69120 Heidelberg, Germany; Center for Pediatric and Adolescent Medicine/Pediatric Neurology and Metabolic Medicine, Center for Rare Disorders, Heidelberg University Hospital, Im Neuenheimer Feld 430, D-69120 Heidelberg, Germany

**Keywords:** Orphan drug act, Orphan drug development, Rare disease, Rare rheumatologic disease

## Abstract

**Background:**

Rare rheumatologic diseases are a heterogeneous group of conditions associated with high morbidity. As a whole group, rare rheumatologic diseases afflict millions of people demanding for effective therapies. Therefore, we analyzed the impact of the US Orphan Drug Act on the development of anti-rheumatic orphan drugs.

**Methods:**

Analysis of the FDA database for orphan drug designations.

**Results:**

In the last three decades, out of 77 orphan drug designations, 14 orphan drug approvals were granted by the FDA for the treatment of rare rheumatologic disorders, i.e. juvenile idiopathic arthritis (*N* = 5), cryopyrin-associated periodic syndromes (*N* = 3), uveitis (*N* = 3), familial Mediterranean fever (*N* = 1), anti-neutrophil cytoplasmic antibody-associated vasculitis (*N* = 1), and xerostomia and keratoconjunctivitis sicca in Sjögren’s syndrome (*N* = 1). Mean time (standard deviation) from designation to approval was 3.9 (2.81) [range 1 … 12] years. Number of FDA-approved small molecules (*N* = 6, 43 %) and biologics (*N* = 8, 57 %) was comparable. Almost every fifth (19 %) orphan drug designation was withdrawn. Despite the rarity of conditions, 13/14 pivotal studies were randomized controlled trials.

**Conclusions:**

Orphan drug development is challenging: thirty years of US orphan drug act supported the development and FDA approval of 14 orphan drug programs with anti-rheumatic compounds for six rheumatologic diseases.

## Background

Rheumatologic diseases are associated with high morbidity leading to reduced quality of life, potentially life-long disability, and premature death. Although rare in individuals, as a whole group rare rheumatologic disorders afflict a large group of people [1, 2]. Furthermore, many rare autoinflammatory conditions, such as systemic lupus erythematodes, dermatomyositis, scleroderma, vasculitis, periodic fever syndromes, nonbacterial osteomyelitis, or uveitis can manifest already in childhood. For example, the prevalence of juvenile idiopathic arthritis (JIA), having its onset before 16 years of age, varies between 3.8 and 400 per 100,000 [1, 3]. Considering the definition for an orphan disease in general as a condition affecting less than 7.5 people in 10.000 or less than 200.000 in the US or less than five in 10.000 in the European Union, orphan drug development is tremendously challenged by small sample sizes [2, 4, 5]. In addition, highly variable manifestations of rheumatologic diseases and onset in childhood complicate drug development. The US Orphan Drug Act was passed in 1983 to stimulate the investment into development of treatments for rare diseases by granting various incentives, such as 7 years’ marketing exclusivity, tax credit for 50 % of clinical trial costs, protocol assistance, Food and Drug Administration (FDA) fee waiver, and orphan grants programs [4]. The impact of the US orphan drug act on successful drug development for the treatment of rare rheumatologic diseases has not been systematically analyzed. We therefore analyzed how many orphan drugs were designated and subsequently approved by the FDA between 1983 and 2013 to treat rare rheumatologic diseases.

## Methods

### Search strategy

For quantitative analysis, we searched the publically available FDA database for orphan drug designations [6] using the following disease entities as search terms (terms for pediatric rheumatologic diseases in alphabetical order [7]): arthritis, antiphospholipid antibody syndrome, Behçet syndrome, collagenosis, CREST syndrome (calcinosis, Raynaud’s phenomenon, esophageal dysmotility, sclerodactyly and telangiectasia), childhood and adolescence arthritis, CAPS (cryopyrin-associated periodic syndromes) such as CINCA/NOMID (chronic infantile neurologic cutaneous and articular syndrome/neonatal-onset multisystem inflammatory disease), MWS (Muckle-Wells syndrome) or FCAS (familial cold autoinflammatory syndrome), cutaneous leukocytoclastic vasculitis, dermatomyositis, diffuse sclerosis, drug induced lupus erythematosus, enthesitis-related arthritis, eosinophilic fasciitis (Shulman syndrome), eosinophilic granulomatosis with polyangiitis (Churg-Strauss syndrome), familial cold autoinflammatory syndrome, familial Mediterranean fever, Felty syndrome, granulomatosis with polyangiitis, Hyper IgD syndrome, idiopathic uveitis, IgA vasculitis (Henoch - Schönlein purpura), infantile sarcoidosis, isolated sacroiliitis, juvenile ankylosing spondylitis, juvenile idiopathic arthritis, Kawasaki syndrome, limited systemic sclerodermia, localized scleroderma, lupus, microscopic polyangiitis, mixed connective tissue disease, non-bacterial osteitis, oligoarthritis, periodic fever syndromes, PFAPA syndrome (periodic fever, aphthous stomatitis, pharyngitis, adenitis), polyarteritis nodosa, polymyositis, primary angiitis of the central nervous system, psoriatic arthritis, relapsing polychronditis, rheumatoid factor negative polyarthritis, rheumatoid factor positive polyarthritis, Sharp syndrome, Sjögren’s syndrome, spondyloarthritis due to chronic inflammatory intestinal disorders, spondyloarthritis due to psoriasis, spondylodiscitis, spondylolisthesis, spondylolysis, systemic arthritis, systemic lupus erythematosus, systemic sclerosis, Takayasu arteritis, transient synovitis, TRAPS (tumor necrosis factor receptor associated periodic syndrome), undifferentiated juvenile idiopathic arthritis, and vasculitis (e.g., Anti-neutrophil cytoplasmic antibody (ANCA) associated vasculitis). Non-English alphabet letters such as umlaut were searched with alternative spellings (e.g., ö was also entered as both o and oe). Acronyms were searched by both the acronym and the full wording. Disease terms with more than one word were searched with the full disease term and with each individual component of the term, e.g., “juvenile idiopathic arthritis” was searched as “juvenile”, “idiopathic”, “arthritis”, and “juvenile idiopathic arthritis”. All search results were subsequently checked for plausibility. All data entries from 1/1/1983 until 12/31/2013 were considered. Original terms from the FDA orphan drug database or FDA label were used to describe designated or approved indications to conform to source data.

### Definitions

Identified compounds were categorized either according to their drug class or pharmacologic category. Within the respective classification, remaining compounds that could not be clearly allocated to a particular category were grouped as others. For classification according to their drug class, the compounds were accounted as small molecules or biologics. A small molecule was defined as a low-weight molecule (typically <1,000 Da) which is usually derived from chemical synthesis and can be fully characterized by analytical techniques [8, 9]. In contrast, biologics are large-molecular weight and structurally complex proteins that are derived from living cells through biotechnological processes (e.g., antibody methods, controlled gene expression or recombinant DNA) [8, 9]. Biologics were further differentiated into antibodies and fusion proteins. For classification according to pharmacologic category, compounds were grouped according to their mechanism of action which was gained from the FDA label for approved compounds. For the assessment of the putative mechanism of action of compounds that were not approved by the FDA, we started from the designated indication and selected the most likely mechanism of action causing the intended effect.

### Statistics

Data were analyzed by standard methods of descriptive statistics with SAS Enterprise Guide version 5.1 (SAS, Cary, NC, USA). Continuous variables are presented as means, standard deviations, and ranges. For categorical variables, N and percentages were calculated. Time-to-approval was calculated as the time span from orphan drug designation to FDA approval.

## Results

### Orphan drug designations

**Fig. 1:**
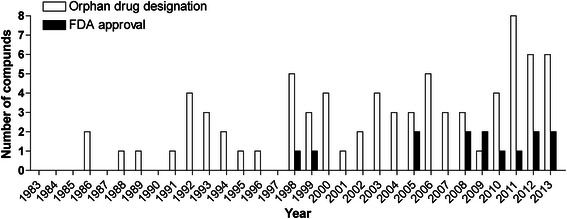
Orphan drugs for rare rheumatologic disorders – number of orphan drug designations (*open box*) and FDA approvals (*black box*) per year

**Fig. 2:**
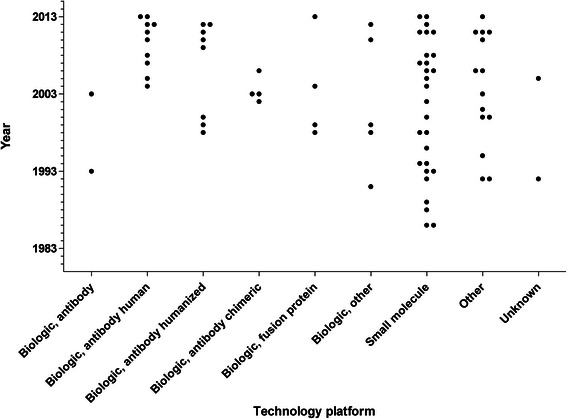
Orphan drugs for rare rheumatologic disorders: year of orphan drug designation by technology platform

Overall, 77 orphan drug designations were granted for 64 different compounds between 1983 and 2013 (Fig. 1, Table 1). The first orphan drug designations were granted in 1986 for two compounds, i.e. guanethidine monosulfate (for the treatment of moderate to severe reflex sympathetic dystrophy and causalgia) and dimethyl sulfoxide (for the treatment of cutaneous manifestations of scleroderma). Both designations were subsequently withdrawn. Most designations per year were granted in 2011 (*N* = 8). When analyzed according to drug classes, 33/77 (43 %) designations were granted for 23 different biologics, 28/77 (36 %) for 27 different small molecules and 14/77 (18 %) designations for others representing 12 different compounds (Table 1). With two designations the drug class of the compounds was unknown (i.e., AI-RSA and Interleukin-1 Trap). Biologics were further differentiated into chimeric antibodies (4/33), human antibodies (10/33), humanized antibodies (8/33), unspecified antibodies (2/33) (i.e., human anti-tumor necrosis factor alpha monoclonal antibody and monoclonal antibody for immunization against lupus nephritis), fusion proteins (4/33), and other biologics (5/33) (i.e., anakinra, hanferon, interferon beta-1a, interleukin-1 receptor antagonist human recombinant, and a DNA plasmid pVGI.1 (VEGF2)) (Fig. 2).

**Table 1:** Drug class and indications of designated anti-rheumatic orphan drugs

Drug class	Compound	Designated indication according to FDA database
Biologic		
Chimeric monoclonal antibody	Infliximab	Treatment of juvenile rheumatoid arthritis. Treatment of giant cell arteritis. Treatment of chronic sarcoidosis.
	Rituximab	For the use in combination with glucocorticoids for the treatment of patients with Wegener’s granulomatosis (WG) and microscopic polyangiitis (MPA).
Human monoclonal antibody	Adalimumab	Treatment of juvenile rheumatoid arthritis.
	Canakinumab	Treatment of pediatric (age 16 and under) juvenile rheumatoid arthritis. Treatment of cryopyrin-associated periodic syndromes. Treatment of Tumor Necrosis Factor-receptor associated periodic syndrome (TRAPS). Treatment of hyperimmunoglobulinemia D and periodic fever syndrome. Treatment of familial mediterranean fever.
	Golimumab	Treatment of chronic sarcoidosis. Treatment of sarcoidosis.
	Secukinumab	Adjunctive treatment of chronic non-infectious uveitis requiring systemic immunosuppression.
	Ustekinumab	Treatment of chronic sarcoidosis.
Humanized monoclonal antibody	Eculizumab	Treatment of dermatomyositis.
	Gevokizumab	Treatment of Behçet disease. Treatment of non-infectious intermediate, posterior or pan uveitis, or chronic non-infectious anterior uveitis.
	Humanized monoclonal antibody to CD40L (IDEC-131)	Treatment of systemic lupus erythematosus.
	Humanized, afucosylated IgG1 kappa monoclonal antibody	Treatment of scleroderma.
	Mepolizumab	Treatment of Churg-Strauss Syndrome.
	Recombinant humanized monoclonal antibody 5c8	Treatment of systemic lupus erythematosus.
	Tocilizumab	Treatment of pediatric patients (age 16 years and younger) with polyarticular-course juvenile idiopathic arthritis.
Unspecified monoclonal antibody	Human anti- Tumor Necrosis Factor alpha monoclonal antibody	Treatment of uveitis of the posterior segment of non-infectious etiology, and uveitis of the anterior segment of non-infectious etiology and refractory to conventional therapy.
	Monoclonal antibody for immunization against lupus nephritis	Treatment of lupus nephritis.
Fusion protein	Etanercept	Reduction in signs and symptoms of moderately to severely active polyarticular-course juvenile rheumatoid arthritis in patients who have had an inadequate response to one or more disease-modifying anti-rheumatic drugs. Treatment of Wegener’s granulomatosis.
	Rilonacept	Treatment of CIAS1-associated periodic syndromes. Treatment of familial Mediterranean fever.
Other	Anakinra	Treatment of cryopyrin-associated periodic syndromes.
	Hanferon	Treatment of Behçet disease.
	Interferon beta-1a	Treatment of juvenile rheumatoid arthritis.
	Interleukin-1 receptor antagonist, human recombinant	Treatment of juvenile rheumatoid arthritis.
	pVGI.1 (VEGF2)	Treatment of thromboangiitis obliterans.
Small molecule	8-methoxsalen	For use in conjunction with the UVAR photopheresis to treat diffuse systemic sclerosis.
	Apremilast	Treatment of Behçet disease.
	Bindarit	Treatment of lupus nephritis.
	Bromhexine	Treatment of mild to moderate keratoconjunctivitis sicca in patients with Sjögren's syndrome.
	Clindamycin hydrochloride	Treatment of sarcoidosis.
	Colchicine	Treatment of familial Mediterranean fever. Treatment of Behçet Syndrome.
	Cyclosporine (ophthalmic)	Treatment of severe keratoconjunctivitis sicca associated with Sjogren’s syndrome.
	Dehydroepiandrosterone	Treatment of systemic lupus erythematosus (SLE) and the reduction in the use of steroids in steroid-dependent SLE patients.
	Dexamethasone (intravitreal implant)	Treatment of non-infectious ocular inflammation of the posterior segment in patients with intermediate, posterior, and panuveitis.
	Difluprednate (ophthalmic solution)	Treatment of endogenous and traumatic anterior uveitis and panuveitis.
	Dimethyl sulfoxide	Treatment of cutaneous manifestations of scleroderma.
	Fluocinolone acetonide (intravitreal implant)	Treatment uveitis involving the posterior segment of the eye.
	Gammalinolenic acid	Treatment of juvenile rheumatoid arthritis.
	Guanethidine monosulfate	Treatment of moderate to severe reflex sympathetic dystrophy and causalgia.
	Gusperimus trihydrochloride	Treatment of Wegener’s granulomatosis.
	Meloxicam	Treatment of juvenile rheumatoid arthritis.
	Methotrexate	Treatment of juvenile rheumatoid arthritis.
	Minocycline hydrochloride	Treatment of sarcoidosis.
	Nabumetone	Treatment of pediatric juvenile rheumatoid arthritis.
	N-acetyl-procainamide	Prevention of life-threatening ventricular arrhythmias in patients with documented procainamide-induced lupus.
	Nitric oxide	Diagnosis of sarcoidosis.
	Pentoxifylline	Treatment of Behçet disease.
	Pilocarpine HCl	Treatment of xerostomia and keratoconjunctivitis sicca in Sjögren's syndrome patients.
	Rofecoxib	Treatment of juvenile rheumatoid arthritis.
	Siponimod	Treatment of polymyositis.
	Sirolimus	Treatment of chronic/refractory anterior noninfectious uveitis, noninfectious intermediate uveitis, noninfectious panuveitis and non-infectious, uveitis affecting the posterior of the eye (NICUPS).
	Thymopentin	Treatment of sarcoidosis.
Other	Abetimus	Treatment of lupus nephritis.
	Allogeneic ex-vivo expanded placental adherent stromal cells	Treatment of thromboangiitis obliterans (Buerger’s disease).
	Bone marrow-derived mononuclear cells	Treatment of thromboangiitis obliterans (Buerger’s disease).
	Cyclo {{(E,Z)-(2S, 3R, 4R)-3-hydroxy-4-methyl-2-(methylamino) nona-6,8-dienoyl}-L-2-aminobytyrl-N-methyl-glycyl-N-methyl-L-leucyl-L-valyl-N-methyl-L-leucyl-L-alanyl-D-alanyl-N-methyl-L-leucyl-N-methyl-L-leucyl-N-methyl-L-valyl}	Treatment and chronic control of non-infectious posterior, intermediate and pan-uveitis.
	Human gammaglobulin	Treatment for juvenile rheumatoid arthritis. Treatment of idiopathic inflammatory myopathies.
	Immune globulin intravenous (IVIG)	Treatment of juvenile rheumatoid arthritis. Treatment of polymyositis/dermatomyositis.
	Kre-Celazine (Oral Buffered Creatine-Cetylated Fatty Acid Compound)	Treatment of juvenile rheumatoid arthritis joint and related tissue inflammation in the pediatric population.
	Lactobacillus brevis CD2	Treatment Behçet disease.
	L-pyr-L-glu-L-gln-L-leu-L-glu-L-arg-L-ala-L-leu-L-asn-L-ser-L-ser	Treatment of neuropathic pain in patients with sarcoidosis.
	Natural human lymphoblastoid interferon-alpha	Treatment of Behçet disease.
	Peptide 144 (TGF beta-1-inhibitor)	Treatment of localized scleroderma.
	Purified type II collagen	Treatment of juvenile rheumatoid arthritis.
Unknown	AI-RSA	Treatment of autoimmune uveitis.
	Interleukin-1 Trap	Treatment of Still’s disease including juvenile rheumatoid arthritis and adult-onset Still’s disease.

### FDA approvals

**Fig. 3:**
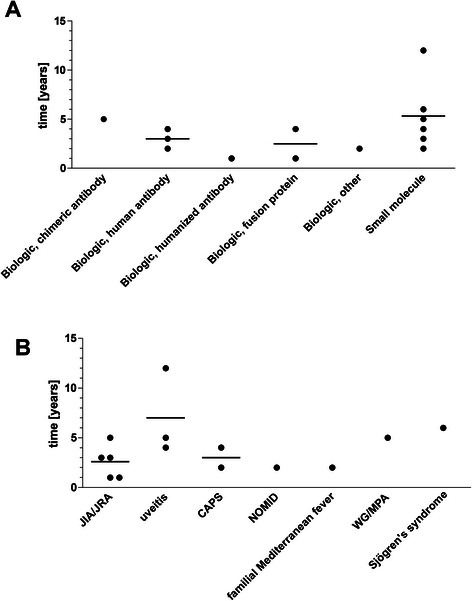
Orphan drugs for rare rheumatologic disorders – time to FDA approval. **a** by drug class. **b** by disease. Lines indicate means

Between 1983 and 2013, 14/77 (18 %) orphan drug designations received FDA approval representing 13 different compounds, because canakinumab was approved for two indications (Table 2, Fig. 1). Five drugs were approved for JIA, three for CAPS, three for uveitis, one for familial Mediterranean fever, one for ANCA associated vasculitis, and one for xerostomia and keratoconjunctivitis sicca in Sjögren's syndrome patients (Table 2). The first approved orphan compound for a rare rheumatologic condition was pilocarpine hydrochloride for treating xerostomia and keratoconjunctivitis in Sjögren’s syndrome in 1998. Mean time (standard deviation) from designation to approval was 3.9 (2.81) [range 1 … 12] years. Divided according to drug classes, 6/14 (43 %) FDA orphan drug approvals comprised small molecules and 8/14 (57 %) FDA orphan drug approvals were biologics. The time between orphan drug designation and time of approval was similar for biologics and small molecules (Fig. 3). Most pivotal trials were randomized controlled trials (Table 2).

**Table 2:** Orphan drugs for the treatment of rare rheumatologic disorders approved by the FDA between 1983 and 2013

Compound	Approved indication according to FDA label	Pharmacologic category	Designation year	Approval year	Pivotal clinical trial					Reference
					Study design	N	Age	Study duration	Main outcome measure	
Pilocarpine hydrochloride	Treatment of dry mouth in patients with Sjögren’s syndrome	Cholinergic agonist	1992	1998	RCT	256	mean 57 years (24 – 85)	12 weeks	Global improvement of dry mouth	[26]
					RCT	373	mean 55 years (21 – 84)	12 weeks	Global improvement of dry mouth	
Etanercept	Reduction in signs and symptoms of moderately to severely active polyarticular-course juvenile rheumatoid arthritis in patients who have had an inadequate response to one or more disease-modifying anti-rheumatic drugs	Tumor necrosis factor-alpha inhibitor	1998	1999	RCT	69	2 – 17 years	7 months	JIA definition of improvement criteria	[10]
Dexamethasone (intravitreal implant)	Treatment of non-infectious uveitis affecting the posterior segment of the eye	Glucocorticoid	1998	2010	RCT	153	n/a	8 weeks	Vitreous haze score and 3-line improvement from baseline in best corrected visual acuity	[27]
Fluocinolone acetonide (intravitreal implant)	Treatment of chronic non-infectious uveitis affecting the posterior segment of the eye	Glucocorticoid	2000	2005	RCT	108	n/a	3 years	Rate of recurrence of uveitis affecting the posterior segment of the study eye	[28]
					RCT	116	n/a	3 years	Rate of recurrence of uveitis affecting the posterior segment of the study eye	
Meloxicam	For relief of the signs and symptoms of pauciarticular or polyarticular course juvenile rheumatoid arthritis in patients 2 years of age or older	NSAID	2002	2005	RCT	n/a	≥2 years	12 weeks	ACR Pediatric 30	[29]
					RCT	n/a	≥2 years	12 weeks	ACR Pediatric 30	
Rilonacept	Treatment of cryopyrin-associated periodic syndromes	Interleukin inhibitor	2004	2008	RCT	47	n/a	24 weeks	CAPS symptom score	[30]
Adalimumab	Treatment of juvenile idiopathic arthritis	Tumor necrosis factor-alpha inhibitor	2005	2008	RCT	171	4 – 17 years	32 weeks	ACR Pediatric 30	[31]
					OL	32	2 – ≤ 4 years or ≥ 4 years weighing < 15 kg	120 weeks	Safety	
Rituximab	For the use in combination with glucocorticoids for the treatment of patients with Wegener’s granulomatosis and microscopic polyangiitis	Selective immunosuppressant	2006	2011	RCT	197	≥15 years	18 months	Birmingham Vasculitis Activity Score for Granulomatosis with Polyangiitis	[16]
Colchicine	Treatment of familial Mediterranean fever	Mitotic poison	2007	2009	Evidence for the efficacy was derived from the published literature.					[32]
					RCT	15	n/a	n/a	n/a	
					RCT	22	n/a	n/a	n/a	
Canakinumab	Treatment of cryopyrin-associated periodic syndromes, in adults and children 4 years of age and older	Interleukin inhibitor	2007	2009	RCT	31	9 – 74 years	8 weeks	Minimal or better for physician’s assessment of disease activity, assessment of skin disease, and serum levels of C-Reactive Protein and Serum Amyloid A	[19]
					OL	n/a	4 – 74 years		n/a	
Canakinumab	Treatment of active systemic juvenile idiopathic arthritis in patients aged 2 through 16 years	Interleukin inhibitor	2008	2013	RCT	84	mean 8.5 years (2 – 20)	4 weeks	Adapted ACR Pediatric 30 and absence of fever	[19]
					RCT	177	mean 8.5 years (2 – 20)	n/a	ACR Pediatric 30	
Difluprednate (ophthalmic solution)	Treatment of endogenous anterior uveitis	Glucocorticoid	2008	2012	RCT	110	n/a	2 weeks	Difference in anterior chamber cell grade	[33]
					RCT	90	n/a	2 weeks	Difference in anterior chamber cell grade	
Anakinra	Treatment of neonatal-onset multisystem inflammatory disease	Interleukin inhibitor	2010	2012	OL	43	0.7 – 46 years	60 months	disease-specific Diary Symptom Sum Score	[34]
Tocilizumab	Treatment of active polyarticular juvenile idiopathic arthritis in patients 2 through 16 years of age	Interleukin inhibitor	2012	2013	RCT	188	2 – 17 years	24 weeks	JIA ACR 30 flare	[20]

*ACR Pediatric 30* American College of Rheumatology Pediatric 30 response, *CAPS* Cryopyrin-associated periodic syndromes, *JIA* Juvenile idiopathic arthritis, *JIA ACR 30 flare* Juvenile idiopathic arthritis American College of Rheumatology 30 flare, *JRA* Juvenile rheumatoid arthritis, *n/a* not applicable or no information is provided in the FDA label, *NSAID* Nonsteroidal anti-inflammatory drugs, *OL* Open label, *RCT* Randomized controlled trial, uncontrolled, *SJIA* Systemic juvenile idiopathic arthritis

### Withdrawn orphan drug designations

Overall, 15/77 (19 %) orphan drug designations (14 compounds) were withdrawn before approval (Table 3). The reasons for withdrawals were not captured in the FDA database.

**Table 3:** Withdrawn orphan drug designations between 1983 and 2013 for compounds intended to treat rare rheumatologic disorders

Withrawn compound	Designation year	Designated indication according to FDA database
Dimethyl sulfoxide	1986	Cutaneous manifestations of scleroderma
Guanethidine monosulfate	1986	Moderate to severe reflex sympathetic dystrophy and causalgia
Bromhexine	1989	Mild to moderate keratoconjunctivitis sicca in patients with Sjögren’s syndrome
Immune globulin intravenous (human)	1992	Juvenile rheumatoid arthritis
Immune globulin intravenous (human)	1992	Polymyositis/dermatomyositis
AI-RSA	1992	Autoimmune uveitis
Methotrexate	1993	Juvenile rheumatoid arthritis
Dehydroepiandrosterone	1994	Systemic lupus erythematosus (SLE) and the reduction in the use of steroids in steroid-dependent SLE patients
Interferon beta-1a	1998	Juvenile rheumatoid arthritis
Humanized MAb (IDEC-131) to CD40L	1999	Systemic lupus erythematosus
Etanercept	1999	Wegener’s granulomatosis
pVGI.1 (VEGF2)	1999	Thromboangiitis obliterans
Infliximab	2003	Giant cell arteritis
Human Anti-tumor Necrosis factor alpha monoclonal antibody	2003	Uveitis of the posterior segment of non-infectious etiology, and uveitis of the anterior segment of non-infectious etiology and refractory to conventional therapy
Golimumab	2004	Chronic sarcoidosis

### Pharmacologic categories of designated orphan drugs

Designated compounds were immunomodulators, such as immunosuppressants, immunostimulants, tolerogens, or compounds with unspecified immunomodulatory properties, Nonsteroidal anti-inflammatory drugs (NSAIDs), expectorants, antibiotics, and angiogenic, antiarrhythmic, diagnostic, parasympathomimetic, and sympatholytic agents.

### Design of pivotal clinical trials and endpoints leading to FDA approval

Only one pivotal trial, i.e. the study of anakinra for the treatment of neonatal onset inflammatory disease, was open label (Table 2). The other 12 approved compounds were studied for 13 indications in randomized controlled trials in which withdrawal designs were common. Colchicine received approval for the treatment of familial Mediterranean fever based on data available in the published literature. Most clinical trials were rather small and involved both children and adults. Studies were either uncontrolled (i.e., anakinra) or controlled with placebo (i.e., colchicine, rilonacept, canakinumab in both indications, etanercept, pilocarpine, adalimumab, and tocilizumab), sham procedure (i.e., dexamethasone intravitreal implant), before after control (i.e., fluocinolone acetonide intravitreal implant), or active control (i.e., meloxicam vs. naproxen, difluprednate ophthalmic solution vs. prednisolone, rituximab vs. cyclophosphamide in a non-inferiority design). Etanercept was studied as add-on therapy to an NSAID and/or prednisone.

## Discussion

In 2013, 77 orphan drug designations for rare rheumatologic disorders were granted by the FDA whereof 14 resulted in FDA approval comprising 13 different substances. Almost every fifth designation was withdrawn, mostly with unknown reasons. However, with etanercept safety reasons may have played a role in withdrawal: etanercept was studied in Wegener’s granulomatosis with a higher incidence of malignancies observed in the etanercept group compared with standard therapy [10, 11]. The success rate to achieve marketing approval in rheumatologic orphan drug development is comparable to overall success rates in orphan drug development, i.e. 14 % [12]. In general, failure of orphan drug applications are mainly attributed to the pivotal clinical trial design (e.g. choice of endpoints and target population), inexperience in orphan drug development of sponsors, and a low level of interaction with the FDA (e.g. protocol assistance) [13]. These factors seem to be interdependent because inexperienced sponsors may choose inadequate pivotal clinical trial designs and may benefit most from FDA protocol assistance. The reasons why compounds were withdrawn or not approved e.g. lack of efficacy, safety issues or due to commercially driven decisions, are not available in the FDA database. Although, this information would be of value for the clinician as it may protect patients from unnecessary exposure to further research and should be made publically available.

By definition, drug development in orphan diseases is challenged by small sample sizes [5]. In addition, disease-specific factors, such as the prevalence, disease class, and scientific output, influence success rates in orphan drug development [14]. Rheumatologic diseases include complex pathomechanisms which complicates identification of potential drug targets. Most of the designated compounds target autoimmune and subsequent inflammatory reactions. Particularly, immunomodulators play a pivotal role in disease modification by modulating various pathophysiologically relevant targets in the inflammatory process e.g., antibodies against specific surface antigens on lymphocytes, glucocorticoids that inhibit transcription of inflammatory cytokines such as interleukins and TNF-alpha, decoy receptors, or antagonists for receptors of inflammatory proteins. In addition, compounds that relieve symptoms associated with rheumatologic diseases were designated as orphan drugs, such as pilocarpine-HCl which increases lacrimal secretion and hence alleviates xerostomia and keratoconjunctivitis sicca in Sjögren’s syndrome patients. Nitric oxide (NO), a biomarker of airway inflammation, received orphan drug designation for the diagnosis of sarcoidosis. However, the diagnostic clinical trial could not detect a difference in exhaled NO levels between patients and controls [15]. All remaining 76 orphan drug designations for rheumatologic conditions were of therapeutic purpose.

Orphan drugs for rheumatologic disease hold indications in other disease areas by targeting pathways also relevant in other conditions. For example, rituximab, which is a selective immunosuppressant targeting CD20 surface proteins on B-lymphocytes, is also approved for treatment of Non-Hodgkin’s Lymphoma and Chronic Lymphocytic Leukemia [16]. In contrast, orphan drugs for lysosomal storage disorders target a specific pathway that is unique for the respective conditions. For instance, although Fabry disease and Gaucher disease are sphingolipidoses, the therapeutic enzyme for Fabry disease (agalsidase alfa or beta) is different to the enzyme replacement therapy in Gaucher disease (recombinant glucocerebrosidase) [17, 18].

The choice for or against an orphan drug development pathway can vary within same disease entity: both tocilizumab and canakinumab are approved for the treatment of systemic juvenile idiopathic arthritis. Of interest, the orphan program with canakinumab consisted of two studies with *N* = 261 participants followed for 4 and 48 weeks, whereas the non-orphan tocilizumab program had only one clinical trial and involved *N* = 112 subjects with a study duration of 12 weeks [19, 20]. Sample size and design of pivotal clinical trials for the approved rheumatologic orphan drug indication varied. Most studies were randomized placebo controlled trials which corroborates that drug development in rare diseases is possible at a high level of evidence. The FDA guidance for industry for the development of drug products for the treatment of rheumatoid arthritis recommends the use of efficacy endpoints capturing clinical remission and prevention of structural damage, suggests to limit the use of placebo both in short-term and long-term trials, and encourages study designs with active comparators [21]. Endpoints in clinical trials of the orphan drugs approved for rheumatologic disorders included categorical variables such as number of attacks in familial Mediterranean fever under colchicine, rate of recurrence of uveitis under fluocinolone acetonid intravitreal implant, disease scores such as vitreous haze score or anterior chamber cell grade for uveitis under difluprednate ophthalmic emulsion or dexamethasone intravitreal implant, multisystem composite disease scores, such as the Birmingham Vasculitis Activity Score for Granulomatosis with Polyangiitis [22] or the American College of Rheumatology Pediatric 30 criteria for improvement which comprises subjective, objective and biochemical components [23]. For the future, it would be desirable to capture the impact of the tested intervention on patients’ needs by including patient centered outcome measures such as the Canadian Occupational Performance Measure as clinical trial endpoints where possible [24]. As signals may not become evident or may not be detectable in the initial small and short clinical trials (Table 2), it is desirable to follow-up on the long-term effectiveness and safety outcome in systematic disease registries after drug approval. Compared to oncological orphan drugs with a shorter time to approval, orphan drug designations in rheumatologic diseases seem to be either set earlier in the drug development process or the process from designation to approval may be slower [12]. In general, orphan drug designations are granted during the final steps of the drug development process [25]. Although, the orphan drug designation is arbitrary in the drug development process, time to approval allows an approximate estimation of orphan drug approvals of currently filed designated orphan drugs.

This quantitative analysis has several limitations. We made the assumption that the designation of a compound as an orphan drug was considered a surrogate for the intent to develop a drug for a disease. However, not all manufacturers may seek orphan drug designation by the FDA and information may therefore not be transparent, e.g. due to patent considerations. The amount of designations might be prone to bias since one compound might be designated as an orphan drug several times, e.g. interleukin 1-trap and rilonacept. The true duration of the development program may not be reflected by time to approval because the time of orphan designation may be arbitrary in the drug development process and, thus does not allowing a comparison between orphan drug development and non-orphan drug development. Data from the European Medicine Agency data were not formally analyzed. We chose this approach, because the European orphan legislation was only introduced in 2000. Therefore, the European database is less comprehensive compared to the one hosted by the FDA. However, drug development for orphan conditions is a global effort. EMA orphan drug designations show similar trends (data not shown). Therefore, the present formal analysis of the FDA data and their impact for patients around the world are considered generalizable.

## Conclusions

In conclusion, orphan drug development is challenging: 30 years of US orphan drug act delivered 14 orphan drug programs with anti-rheumatic or supportive compounds for six rare rheumatologic conditions. Although, rarity and diversity of conditions account for the small sizes all but one pivotal study were randomized controlled trials.

## Abbreviations

ANCA: anti-neutrophil cytoplasmic antibody
CAPS: cryopyrin-associated periodic syndromes
CINCA: chronic infantile neurologic cutaneous and articular syndrome
CREST syndrome: calcinosis, Raynaud's phenomenon, esophageal dysmotility, sclerodactyly, and telangiectasia
DNA: desoxyribonucleic acid
FCAS: familial cold autoinflammatory syndrome
FDA: Food and Drug Administration
IgA: immunglobulin A
IgD: immunglobulin D
JIA: juvenile idiopathic arthritis
MWS: Muckle-Wells Syndrome
NO: nitric oxide
NOMID: neonatal-onset multisystem inflammatory disease
NSAID: nonsteroidal anti-inflammatory drug
PFAPA syndrome: periodic fever, aphthous stomatitis, pharyngitis, adenitis
TRAPS: tumor necrosis factor receptor associated periodic syndrome
VEGF2: vascular endothelial growth factor 2
